# BCL-2 family isoforms in apoptosis and cancer

**DOI:** 10.1038/s41419-019-1407-6

**Published:** 2019-02-21

**Authors:** Chloe F. A. Warren, Michelle W. Wong-Brown, Nikola A. Bowden

**Affiliations:** 1 Faculty of Health and Medicine, School of Biomedical Sciences and Pharmacy, University of Newcastle, Newcastle, NSW Australia; 2 Hunter Medical Research Institute, Newcastle, NSW Australia; 3 Faculty of Health and Medicine, School of Medicine and Public Health, University of Newcastle, Newcastle, NSW Australia

## Abstract

The BCl-2 family has long been identified for its role in apoptosis. Following the initial discovery of BCL-2 in the context of B-cell lymphoma in the 1980s, a number of homologous proteins have since been identified. The members of the Bcl-2 family are designated as such due to their BCL-2 homology (BH) domains and involvement in apoptosis regulation. The BH domains facilitate the family members’ interactions with each other and can indicate pro- or anti-apoptotic function. Traditionally, these proteins are categorised into one of the three subfamilies; anti-apoptotic, BH3-only (pro-apoptotic), and pore-forming or ‘executioner’ (pro-apoptotic) proteins. Each of the BH3-only or anti-apoptotic proteins has a distinct pattern of activation, localisation and response to cell death or survival stimuli. All of these can vary across cell or stress types, or developmental stage, and this can cause the delineation of the roles of BCL-2 family members. Added to this complexity is the presence of relatively uncharacterised isoforms of many of the BCL-2 family members. There is a gap in our knowledge regarding the function of BCL-2 family isoforms. BH domain status is not always predictive or indicative of protein function, and several other important sequences, which can contribute to apoptotic activity have been identified. While therapeutic strategies targeting the BCL-2 family are constantly under development, it is imperative that we understand the molecules, which we are attempting to target. This review, discusses our current knowledge of anti-apoptotic BCL-2 family isoforms. With significant improvements in the potential for splicing therapies, it is important that we begin to understand the distinctions of the BCL-2 family, not limited to just the mechanisms of apoptosis control, but in their roles outside of apoptosis.

## Facts

- BCL-2 family members play an integral role in apoptosis, but also contribute to many other cellular functions.
- Isoforms of almost all of the BCL-2 family members have been identified and some are well characterised.
- Therapeutics targeting BCL-2 show great promise for the treatment of cancer.

## Open questions

- What is the functional role of uncharacterised BCL-2 family member isoforms in apoptosis and normal cellular functions, in particular the BCL-2 isoform BCL-2β?
- Is the presence and varied functional characteristics of BCL-2 family isoforms being considered in the development of therapeutics targeting BCL-2?
- Is there potential to target BCL-2 family member isoforms that are expressed higher in cancer?

## Introduction

The BCl-2 family has long been identified for its role in apoptosis. Following the initial discovery of BCL-2 in the context of B-cell lymphoma in the 1980s, a number of homologous proteins have since been identified^1–3^. The members of the Bcl-2 family are designated as such due to their BCL-2 homology (BH) domains and involvement in apoptosis regulation. The BH domains facilitate the family members’ interactions with each other, and can indicate pro- or anti-apoptotic function^4,5^. Traditionally, these proteins are categorised into one of three subfamilies; anti-apoptotic, BH3-only (pro-apoptotic), and pore-forming or ‘executioner’ (pro-apoptotic) proteins. Subfamily categorization has been traditionally based on BH and transmembrane domain and anti- or pro-apoptotic function status, as well as pore-forming ability (as shown in Table 1).

**Table 1:** BCL-2 subfamilies and members

Subfamily	Activity	BH Domain Status	Members
Anti-apoptotic	Anti-apoptotic	Presence of BH4 domain	BCL-2 BCL-X_L_ BCL-W BCL-B (BCL2L10) MCL-1L
		Absence of BH4 domain	MCL-1 BFL-1/A1 BCL2L12^13^
Pore- forming executioners	Pro-apoptotic	Multi-domain	BAX BAK^104^ BOK^105^
BH3-only	Pro-apoptotic	Activator–binds to pro-apoptotic and anti-apoptotic Bcl-2 multiregion proteins^13^	BIM BID Puma Mule^13,106^
		Sensitizer–displaces activator BH3-only proteins from anti-apoptotic proteins to promote apoptosis^13^	BAD Noxa BIK./BLK BMF HRK/DP5 Beclin-1
	Potential pro-apoptotic		BCL-Rambo (BCL2L13)^107^ BCL-G (BCL2L14)^107^ MCL-1S^108^ MCL-1ES^108^

The role of the BCL-2 family in apoptotic regulation is typically described as the anti-apoptotic and pro-apoptotic BH3-only members existing in a state of competitive flux to influence the activation of the pore-forming executioners^6,7^. The ratio of pro- to anti-apoptotic subfamily members present in a cell can be altered by a number of signalling pathways, effectively relaying information on cellular stress, such as available nutrients, DNA damage, and protein processing^8^. Once the executioners are activated, the molecules come together to form pores in the outer mitochondrial membrane (MOM) and thus trigger mitochondrial outer membrane permeability (MOMP), and therefore apoptosis^9–11^.

The BH domains are considered central to subfamily categorization as they facilitate the interaction of family members. BH3 was initially highlighted as an important domain as it was demonstrated to be vital for the interaction of the anti-apoptotic BCL-X_L_ and the executioner BAK, as well as for its apoptotic activity. The BH3 domain is vital for the correct folding of a hydrophobic pocket, within which BCL-2 members can interact^12,13^. Consequently, point mutations or deletions of the BH3 domain have been shown to significantly reduce the pro-apoptotic activity of a number of BH3-only proteins^14^. The BH4 domain is thought to be similarly significant for the anti-apoptotic subfamily; deletion of the BH4 domain can switch function to pro-apoptotic, while retention of the BH4 domain alone is sufficient to block changes in mitochondrial potential^14^.

Beyond this understanding of a competitive flux, there are several hypotheses regarding how the BCL-2 family members interact, including direct and indirect interactions amongst family members (summarised in Supplementary Table 1). Each of the BH3-only or anti-apoptotic proteins have patterns of activation, localisation and response to specific death or survival stimuli. Binding selectivity between members of the different classes of BCL2 proteins also varies, for example, some BH3-only proteins bind non-specifically to several BCL2 prosurvival proteins while others tend to bind in a more specific manner. Similarly, BCL2 prosurvival family members can selectively bind to and limit activity of BAX or BAK. All of these interactions can vary across cell or stress types, or developmental stage, and this can cause the delineation of the roles of BCL-2 family members. Added to this complexity is the presence of relatively uncharacterised isoforms of many of the BCL-2 family members.

### BCL-2 anti-apoptotic subfamily

This review focuses on the BCL-2 anti-apoptotic subfamily and known isoforms. Traditionally, members of this family are identified by their anti-apoptotic activity as well as the presence of BH4 and transmembrane domains for anchoring to cellular membranes^15^. Some members of this anti-apoptotic subfamily lack some of these physical features, have isoforms translated from the same gene which actually have pro-apoptotic activity, or can have their activity modulated by post-translational modification, as reviewed below and summarised in Table 2.

**Table 2:**
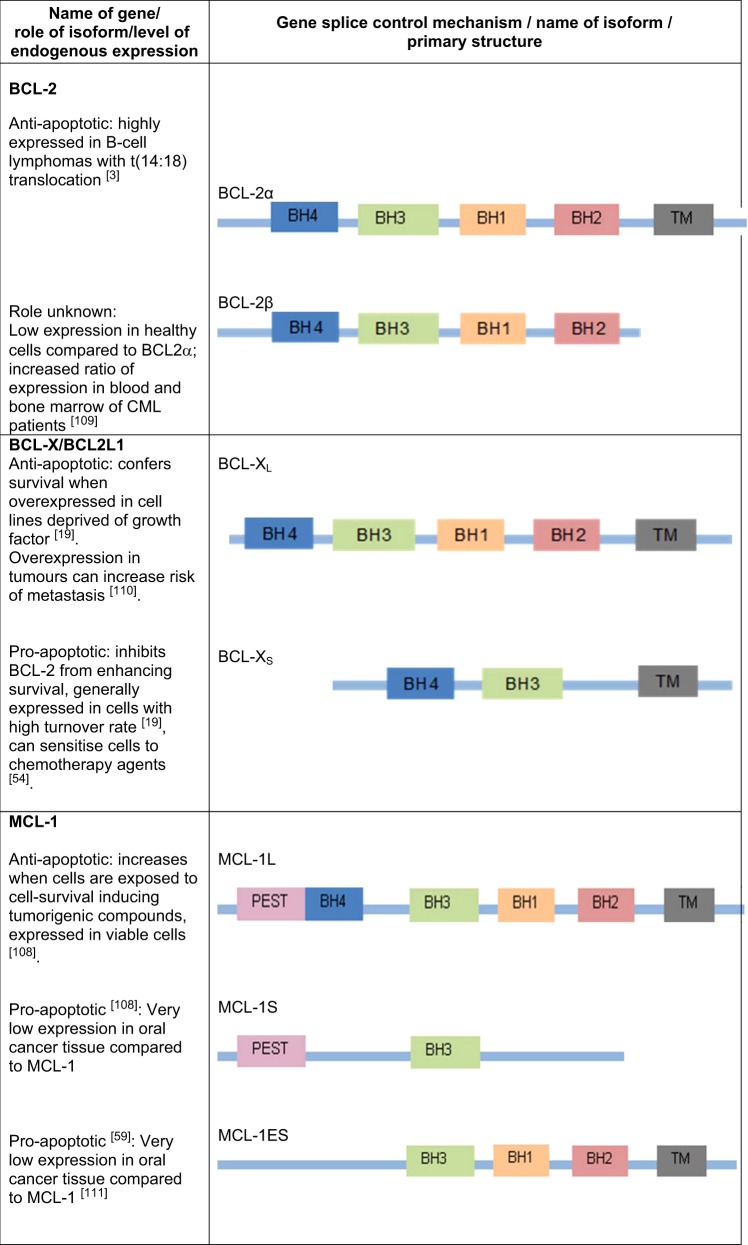

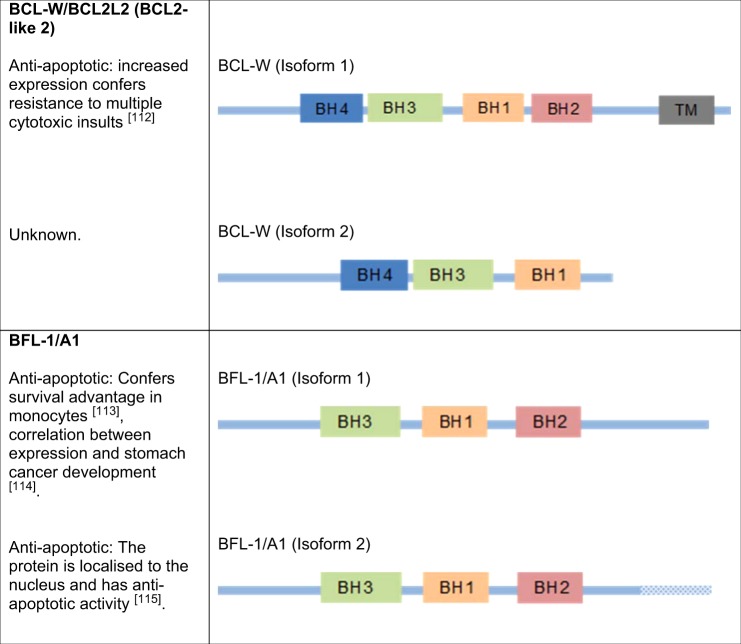
Roles within the anti-apoptotic Bcl-2 subfamily^3,19,54,59,108–115^

### BCL-X

The BCL-X or BCL2L1 (BCL2-like 1) gene has 44% homology to BCL-2. It has two well-known isoforms, BCL-X_L_ and BCL-X_S_ (Table 2), as well as a number of other characterised isoforms^16,17^. The two major isoforms arise from alternative splicing of BCL-X; splicing at the distal end of the 5′ splice site within the first coding exon for production of BCL-X_L_ and at the proximal end for BCL-X_S_. Interestingly, the two isoforms have a different role in apoptosis. While BCL-X_L_ is anti-apoptotic, BCL-X_S_ is pro-apoptotic. Overexpression of BCL-X_L_, but not BCL-X_S_, confers survival in IL-3-dependent cells following IL-3 deprivation^18^. Transfection of these IL-3-dependent cells with BCL-X_S_ reinstates their sensitivity to IL-3 removal, regardless of levels of anti-apoptotic BCL-2^19^. This protective effect has been seen in several cell types, in response to chemotherapeutic drug treatment and growth factor removal^20^.

The BCL-X_L_ protein is comprised of seven alpha-helices, where the two central hydrophobic helices (α5 and α6) are surrounded by five amphipathic helices (α3, α6, α1, α2, and α7). The BH1, BH2, and BH3 domains sit in close proximity, and form a hydrophobic cleft for binding other family members. The C-terminal transmembrane domain extends from the α7 helix. The N-terminal helix (α1) is essential for maintenance of structure stabilisation as it forms extensive interactions with the other helices. The BH3 domain is contained within the α2 helix, the BH1 domain across the α4 and α5, and BH2 across α6 and α7^21^.

The protein structure of BCL-X_S_ has not been comprehensively described, but the loss of both BH1 and BH2 domains would significantly alter the hydrophobic binding cleft^22^. While BCL-X_L_ exerts its anti-apoptotic regulation by formation of heterodimers with both BAX and BAK, the pro-apoptotic function of BCL-X_S_ is derived from its capacity to disrupt the BAK/VDAC complex through its interaction with voltage-dependent anion channel (VDAC), thus freeing BAK for activation^23^. This highlights the difference in binding capacity between the two isoforms.

Since this discovery of the alternate functions of the two variants, the mechanisms of splicing control of the BCL-X gene have been a matter of some interest. It has been demonstrated that switching of splicing favour is induced by cellular stress, specifically DNA damage^24,25^, protein synthesis stalling^26^, and protein kinase C inhibition^27,28^. The induction of generic cellular stress via treatment with the drug ceramide as well as the combination of epigallocatechin-3-gallate (EGCG) and non-steroidal anti-inflammatory (ibuprofen) have also been shown to shift splice favour^29,30^.

Immunoprecipitation assays on known regulatory regions have identified proteins essential to BCL-X splice control, such as HNRNPK and PTBP1^31,32^. In addition, investigation of known pro-apoptotic transcription factor targets (SC35 via E2F1)^25^, RNA binding proteins (SAM68, SAP155)^33,34^, splice modulators (hnRNP F/H, SRp30c, RBm25, Akt SUMO, TCERG1)^35–39^, and pathways demonstrated to shift Bcl-Xs splicing favour (SB1 and RBM25)^24,37^, have identified a number of other spliceosomal and RNA binding proteins which are involved in BCL-X splicing regulation. As well as these proteins, a lncRNA, RNA INXS, transcribed from the opposite strand of BCL-X, and proto-oncogenes FBI-1 and FYN, are capable of modulating SAM68 activity in favour of BCL-X_S_ splicing^40,41^.

BCL-X_L_ is also known to be involved in calcium signalling regulation via IP3R and VDAC1^42,43^, can regulate Ca^2+^ homeostasis when localised at the endoplasmic reticulum^44^ and reduce mitochondrial Ca^2+^ uptake^45^. The RNA-binding protein HuR, a translational repressor of BCL-X_L_, can also affect maintenance of mitochondrial morphology, which regulates cellular apoptosis, through translational control of BCL-X_L_ expression^46^. BCL-X_L_ has been linked to non-apoptotic cell death by binding the tumour suppressor Beclin 1, subsequently inhibiting autophagy^47^. The overexpression of BCL-X_L_ has been shown to protect endothelial cells from TNF-mediated apoptosis and is involved in inflammatory response by inhibiting the activation of NF-κB and thus the upregulation of proinflammatory genes^48^. Interestingly, BCL-X_L_ has also been shown to have apoptosis-independent function in metastasis in pancreatic neuroendocrine tumour and breast cancer cell lines via nuclear promotion of epithelial–mesenchymal transition, migration, invasion and stemness^49^ and in chemoresistance via RAS interaction and influence on EMT and regulation of cancer-initiating cell (CICs)^50^.

Interestingly, despite the mass of research conducted on BCL-X_L_ and BCL-X splicing control, there is relatively little known about BCL-X_S_. It is a BAK-dependent pro-apoptotic protein^23,51,52^, but any roles outside of apoptosis regulation have not yet been identified. Induction of an increased ratio of BCL-X_S_ to BCL-X_L_ or overexpression of BCL-X_S_ in cancer cell lines have been shown to have a pro-apoptotic effect^20,53–55^.

### MCL-1

MCL-1 (myeloid leukemia sequence 1) was initially discovered due to its upregulation in the MC-1 hematopoietic cell line during the differentiation from monocyte to macrophage^56^. At the time of discovery, the MCL-1L transcript was the only known transcript, and it was rapidly designated as anti-apoptotic after overexpression was observed to protect cells from heat shock^57^. However, there are now three known isoforms of the gene; MCL-1L, MCl-1S and MCL-1ES (Table 2). Similar to the BCL-X isoforms, the three proteins have different roles in the regulation of apoptosis; MCL-1L is anti-apoptotic, while MCL-1S and MCL-1ES are both pro-apoptotic^58,59^.

The C-terminal domain of MCL-1L is 350 amino acids long and has sequence homology with BCL-2. A central hydrophobic helix (α5) is surrounded by a set of amphipathic helices, which pack tightly against it (α1, α2, α3, α4, α5, α6, α7), where α3 and α4 are less densely packed, and BH1 is contained within α5 and α6. Helices α2, α3 and α4 form the characteristic hydrophobic binding groove and contain the BH3 domain, where α5 and α8 form the base of the groove^60^. MCL-1L also harbours a C-terminal transmembrane domain^58,60^. Unlike other members of the BCL-2 family, the MCL-1L N-terminus contains a PEST sequence that is associated with rapidly degrading proteins, as well as multiple sites for phosphorylation and caspase cleavage sequences^61^. These post-translational modifications can change protein stability and function, and consequently, MCL-1L has a high rate of turnover within the cell and its degradation can be modulated at several points along the N-terminus^58,62^.

Alternatively, skipping of the second exon of the MCL-1 gene gives rise to the 271 amino acid MCL-1S (Table 2). This variant retains the BH3, BH4 and PEST domains, but not the BH1, BH2 and transmembrane domains. This gives rise to an isoform with features characteristic of a BH3-only protein, in which heterodimerization with anti-apoptotic MCL-1L can block its pro-apoptotic activity. In addition, MCL-1S is incapable of binding with BAX, BAK and BIM, whereas BCL-X_L_ interacts strongly with these family members^58^. Besides this initial study that described the key features of MCL-1S, there is relatively little known about the protein.

A third isoform, MCL-1ES, has also been identified. MCL-1ES occurs as a result of alternative splicing within the first exon at a non-canonical donor-acceptor site. The resultant protein is 197 amino acids long and lacks the PEST sequence and BH4 domain present in the other MCL-1 isoforms (Table 2). This isoform displays a pro-apoptotic function, with overexpression of this isoform resulting in decreased resting cell viability and mitochondrial integrity, all leading to cell death^59^. Interestingly, the effects are amplified when MCL-1ES is co-transfected with MCL-1L, an anti-apoptotic family member^59^. Further work has demonstrated that MCL-1ES localisation to the mitochondria and consequent pro-apoptotic activity is dependent on its heterodimerization with MCL-1L^59,63^. Interestingly, the effect of overexpression on apoptosis is BAX/BAK-dependent, and preliminary studies indicate that MCL-1ES can form the mitochondrial pores for the initiation of apoptosis by the release of cytochrome c and activation of MOMP. This activity is dependent on the BH3 domain of MCL-1ES^63^.

Although studies have been performed on splicing control between MCL-1L and MCL-1S, the mechanisms of MCL-1ES splicing control are still unknown. Much of the work in delineating MCL-1 splicing regulators was performed in parallel with BCL-X investigations. For example, treatment of prostate cancer cell lines with EGCG/ibuprofen switched splicing favour to the pro-apoptotic variant for both MCL-1 and BCL-X in a protein phosphatase 1 (PP1)-dependent manner^30^. In addition, a study by Moore et al. (2010) identified that the splicing regulator ASF/SF2, and protein kinases PLK1 and WEE1, can shift splicing in favour of MCL-1S^64^. The same study identified SAP155 as a driver for transcription of pro-apoptotic splice variants for both MCL-1 and BCL-X, and this result has since been validated by other studies^64,65^. These data indicate that MCL-1 splicing regulation is associated with cell cycle control.

Of the three isoforms, only MCL-1L has been found to have roles outside of apoptosis. Like BCL-2 and BCL-X_L_, MCL-1L can regulate autophagy, mitochondrial morphology, and calcium signalling via its interaction with IP3R^66^, and is involved in cell cycle control^61^ and lipid metabolism^67^.

### BCL-2

**Fig. 1:**
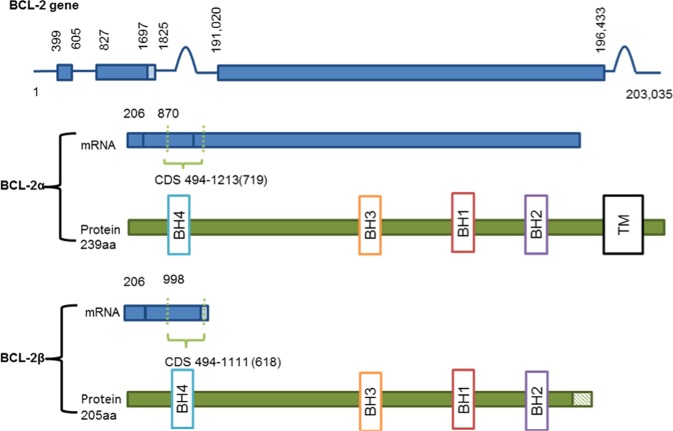
Schematic diagram of BCL-2. Comprised of three exons, with the first two exons encoding the four BH domains and exon 3 encoding the transmembrane domain, with BCL-2β lacking the transmembrane domain

BCL-2 was the first member of the family to be identified, due to its role in B-cell lymphoma. A chromosomal translocation between chromosomes 14 and 18 in this disease, t(14:18), causes enhancement of BCL-2 transcription, which confers a survival advantage to the cancerous cells^1–3^. The BCL-2 gene is comprised of three exons; the first two exons encode the four BH domains, whereas the exon 3 encodes the transmembrane domain that anchors the protein to intracellular membranes^68,69^ (Fig. 1). There are two isoforms of BCL-2; BCL-2α and BCL-2β. While BCL-2α is anti-apoptotic^3,70–72^, BCL-2β is yet to be fully characterised. It lacks exon 3 and thus the transmembrane-anchoring domain, but otherwise shares the same BH domains and general structure of BCL-2α  (Fig. 1). BCL-2β also has an isoform-specific 9-amino acid stretch at its C-terminal domain^73^ (Table 2).

### Roles of BCL-2α

The structure of the BCL-2α protein is similar to BCL-X_L_, with two central hydrophobic helices (α1 and α2) surrounded by five α-helices, and a C-terminal transmembrane domain. Like BCL-X_L_, this characteristic hydrophobic groove is comprised of helices 3, 4, 5 and 6. The structure of the BCL-2β protein is yet to be ascertained, but is known to lack the transmembrane domain, although the significance of this is unclear. While several studies have concluded that a C-truncated BCL-2α is incapable of localising to appropriate organelles, bind target proteins or regulate apoptosis^74–78^, others have disputed the significance of a transmembrane domain^79–81^. However, it is important to note that all these studies have been performed on truncated BCL-2α but not on wildtype BCL-2β.

BCL-2α binds to BAX via its BH1 and BH2 domains, and this interaction is central to its role in apoptosis regulation, as demonstrated in cell lines in response to cellular stress^5^. However, in several models of stress, such as gamma irradiation, IL-3 deprivation of dependent cells, glucocorticoid and cytotoxic drug treatment, and heat shock, it has been shown that, where BCL-2 is upregulated and capable of binding BAX, the capacity of cells to undergo apoptosis is reduced^3,70–72^. These investigations have emphasised the significance of the BH domains.

Like MCL-1L and BCL-X_L_, BCL-2α is the most extensively studied isoform and is involved in autophagy via interaction with Beclin 1, as well as calcium signalling, and has roles outside of apoptosis regulation^82,83^. Interestingly, the interaction between BCL-2 and Beclin 1 occurs at the same site as BH3-only proteins and so competition for the site exists between these proteins^84^. It has also been implicated in DNA repair, including nucleotide excision repair, base excision repair, mismatch repair and double-strand break repair^82,85–87^. In addition, BCL-2α can regulate a number of major transcription factors, including p53^88^, NF-κB, AP1, CRE and NFAT^77^. The different roles of BCL-2α are summarised in Table 3.

**Table 3:** Alternative roles for BCL-2α, other than apoptosis

Cellular process	Description of feature	Role of Bcl-2
Autophagy	Autophagy is a survival mechanism resorted to during starvation, wherein intracellular contents can be recycled for nutritional value.	BCL-2α is capable of inhibiting autophagy via its interaction with Beclin-1, although only when localised at the ER membrane^116^
Apoptosis via p53	p53 is a major tumour suppressor.	BCL-2α can prevent p53 from up regulating pro-apoptotic genes. Interestingly, p53 can also negatively regulate the BCL-2 protein^88,117^
Transcription factor control	Transcription factors regulate gene expression.	BCL-2 can regulate the transcription factors NF-κB, AP1, CRE and NFAT by blocking them from entering the nucleus
Regulation of Ca^2+^ at the endoplasmic reticulum	The ER is the central storage centre for Ca^2+^, a major cellular signalling molecule.	BCL-2 is capable of modulating the activity of IP3R (a Ca^2+^ channel)^42^
Nucleotide excision repair (NER)	NER repairs bulky, helix distorting DNA damage induced by UV irradiation.	Overexpression of BCL-2 attenuates cyclobutane pyrimidine dimer (CPD) removal and the stalling of DNA replication following exposure to UV light^82^
Base excision Repair (BER)	BER occurs throughout the cell cycle to repair non-helix distorting lesions, such as mismatched or damaged single bases.	Overexpression of BCL-2 downregulates BER via APE1 blockage^85,118,119^
Mismatch repair (MMR)	MMR repairs bases which have been mis-incorporated during DNA replication and recombination.	BCL-2 can inhibit MMR via its direct interaction with MSH2^86,120^
Double-strand break repair (DSBR) and non-homologous end joining (NHEJ)	NHEJ is a mechanism of DSBR that rejoins short DNA overhangs (microhomologies) on the ends of either strand of the broken DNA.	Cells with higher expression of BCL-2 had lower levels of end joining and vice versa. This was thought to be due to the ability of BCL-2 to interact with KU proteins, which form a molecular scaffold for the DSBR machinery^87,121^. BCL-2 can also regulate DSBR via its interaction with BRCA1^89^
DSBR and single-strand break repair (SSBR) via PARP1	PARP1 is involved in SSBR and DSBR.	BCL-2 can relocate to sites on the chromatin, where it can directly interact with and inhibit PARP1. This interaction can be disrupted by BH3-only BCL-2 family members (and BH3 mimetic drugs)^122^

### Current evidence for a role of BCL2β

Despite the accumulation of evidence for the many roles of BCL-2α, there has been very little investigation into the role of BCL-2β. Functional protein studies on BCL-2 primarily focuses on the wildtype BCL-2α. Where the BCL-2β isoform is addressed, a recombinant version of a C-terminal-truncated BCL-2α is used for characterisation studies and it has been assumed this structure shares the same function as BCL-2β, as both lack the transmembrane domain^73^. Characterisation studies on the BCL-2β isoform have so far been limited to cloned versions of the genes, which do not accurately reflect the naturally-occurring sequence^74,76,78,89^.

**Fig. 2:**
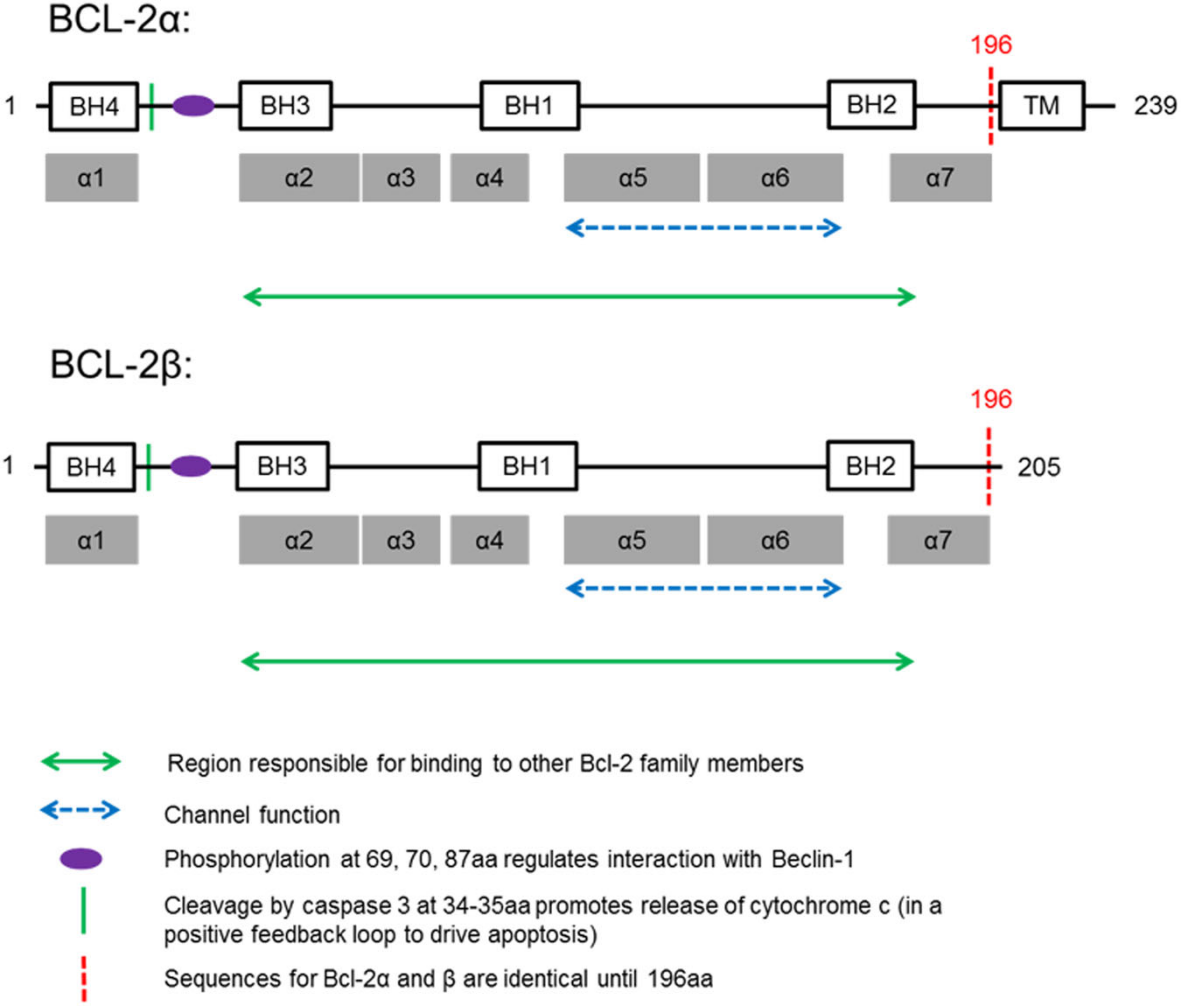
Primary protein structures of BCL-2α and BCL-2β. This figure is based on experiments on BCL-2 and the highly homologous BCL-X_L_. It illustrates the similarities between the isoforms. BH1, BH2, and BH3 are required for heterodimerisation with BCL-2 family members^5,21,123^. Channels are formed by α-helices 5 and 6^124^. Phosphorylation by MAPK8 (mitogen-activated kinase 8) at specific residues between BH4 and BH3 can modify binding to Beclin-1^125^. Caspase-3 cleavage at amino acids 34–35 can abrogate protein function^126^. The two proteins are identical up to amino acid 196, where they start to differ, with BCL-2β lacking a transmembrane domain and having a specific C-terminal 9-amino acid sequence^73^. This figure was adapted from Belka and Budach (2002)^127^

Studies that have assessed the significance of the transmembrane domain on the capacity for BCL-2 to regulate apoptosis and p53 and to interact with BAX and BRCA1 concluded that the domain was vital for the efficiency of BCL-2 in these roles^74,76,78,90^. In contrast, studies that have concluded that the transmembrane domain is not essential for function were focused on the separate steps of apoptosis activation and/or apoptosis across different cell types^79,80^. Despite these inconsistencies in the literature, it is important to note that C-terminal-truncated BCL-2α does not accurately represent BCL-2β, due to the isoform’s specific 9-amino acid C-terminal sequence (see Fig. 2).

### Targeting the BCL2 family for therapeutic purposes

As the BCL-X and BCL-2 families have essential roles in apoptotic regulation and were initially discovered in the cancer setting, they have garnered interest as therapeutic targets. Several studies have tried to regulate apoptosis using retroviral systems^91^, alteration of localisation apparatus^92^, activity-blocking antibodies^93^, RNAi^94^ and miRNAs^95^. However, the most successful method so far for targeting the BCL-2 family has been through BH3-mimetic molecules.

### BH3-mimetics

Initial proof-of-concept studies that highlighted the potential of BH3-mimetics showed that small molecules which bound to the hydrophobic groove of BCL-X_L_ could block anti-apoptotic function^96^. Since then, many different BH3-mimetics have been developed, and these are summarized in Table 4.

**Table 4:** BH3-mimetics and their targets

Inhibitor	Detail for development	Molecular targets	Stage of clinical trials
ABT-737	Lead compound. Mimics BH3 domain of BAD^128^	BCL-2, BCL-X_L_, BCL-W	–
ABT-263 (Navitoclax)	Based on ABT-737 but has longer half-life and is orally bioavailable^129^	BCL-2, BCL-X_L_, BCL-W	Phase 1/2^130–133^
ABT-199 (Venetoclax)	Derivative of ABT-263^134^	BCL-2	Approved for use in chronic lymphocytic leukaemia (CLL) patients with 17p deletion^135^
S55746	Synthetic small molecule and orally available. BCL-2 selective, and no significant binding to BFL-1 and MCL-1 were observed^136^	BCL-2	Phase 1 (NCT02920697, NCT02920541, NCT02603445)
WEHI-539	Small molecule derived from hydrazinylbenzothiazole cores^137^	BCL-X_L_	Preclinical^138^
A-1155463	Small molecule, more potent and chemically-stable than WEHI-539^139^	BCL-X_L_	Preclinical^139^
A-1331852	Small molecule, orally bioavailable^139^	BCL-X_L_	Preclinical^139, 140^
A-1210477	Derivative of indole-2-carboxylic acid core. Has high affinity to MCL-1, and synergizes with navitoclax to induce apoptosis in multiple cancer cell lines^99^	MCL-1	Preclinical^141, 142^
S63845	Synthetic small molecule inhibitor. Higher affinity for human MCL-1 compared to A1210477^101^ Acts synergistically with ABT-199^143^	MCL-1	Phase 1 (NCT02979366)^144–150^

Several studies have highlighted the significance of low MCL1 expression conferring sensitivity to BH3-mimetics in cell lines^97,98^. MCL-1L is one of the most potent of the BCL-2 family as it has a significantly high affinity with pro-apoptotic members. Several molecules with the potential for binding MCL-1L have been developed, A-1210477 was an early molecule proposed to act directly on MCL-1L to promote apoptosis in cell lines^99,100^. More recently, S63845 a small molecule that binds with high affinity to the BH3-binding groove of MCL1 has been shown to kill MCL1-dependent cancer cells, including multiple myeloma, leukaemia and lymphoma cells^101^.

### Manipulation of splicing

Splicing alters the function of BCL-2 members, therefore there is potential to target this therapeutically by manipulation of gene splicing to favour pro-apoptotic transcripts. Introduction of splicing-switching oligonucleotides that alter BCL-X splicing from BCL-X_L_ to the pro-apoptotic BCL-X_S_ in melanoma cell culture and tumour xenografts was shown to reduce tumour load^102^. Additionally, the transcription factor FBI-1 has been shown to have a role in alternative splicing by interacting with splicing factor SAM68, thus reducing binding of SAM68 to BCL-X and resulting in the preferential splicing of anti-apoptotic BCL-X_L_^41^. The silencing of FBI-1 expression restores the ability of SAM68 to induce splicing of pro-apoptotic BCL-X_S_^41^.

Another way to manipulate splicing is by targeting SAP155, a splicing factor which acts on MCL-1 and BCL-X. Inhibition of this protein by meaymycin B, and potent inhibitor of SAP155, has been used to switch splicing in favour of pro-apoptotic MCL-1s in cell culture^65^. Interestingly, the combination of meaymycin B with BH3-mimetic ABT-737 also induces apoptosis^65^. The activity of SAP155 has also been successfully downregulated using an anti-SAP155 antibody, which induced an increase in the pro-apoptotic BCL-X_S_ isoform compared to BCL-X_L_, and this method can be used to prime the cell for response to apoptosis-inducing treatment^33^.

Targeting splicing factors to favour the expression of pro-apoptosis isoforms is appealing but the non-specific nature of splicing factors will need to be addressed for this to be a superior target than BH3 mimetics. A more targeted approach to manipulating splicing is the use of specific antisense oligonucleotides. Antisense oligonucleotides designed to knock-down exon 2 in MCL-1 pre-mRNA can shift splicing pattern from MCL-1L to MCL-1S^103^. This increases the expression of pro-apoptotic MCL-1S and reduces the level of anti-apoptotic MCL-1L, and was shown to induce apoptosis in basal cell carcinoma and gastric adenocarcinoma cell lines^103^. Manipulation of splicing remains an area of interest that requires further development to be a targeted as a treatment with clinical potential.

### Summary

The BCL-2 family is involved in the regulation of apoptosis and therefore plays a vital role in protecting against cancer. Targeting the apoptotic pathway directly is a valid option for improving or developing new chemotherapies, but it is imperative that we understand the molecules, which we are attempting to modify, manipulate or mimic. As demonstrated in this review, there are gaps in knowledge regarding isoforms of anti-apoptotic BCL-2 family isoforms. Further studies focusing on understanding the variety of splice variants and isoforms and their biological role in apoptosis is required for targets of this pathway to reach their full potential.

## Supplementary information

Supplementary Table Supplementary Table 1
