# Supplementary material for: BCL-2 family isoforms in apoptosis and cancer
Source: Cell Death Dis. 2019 Feb 21;10(3):177. doi: 10.1038/s41419-019-1407-6 (PMC6384907; doi:10.1038/s41419-019-1407-6)
Supplement: Supplementary file 1 — Supplementary Table Supplementary Table 1 [file 41419_2019_1407_MOESM1_ESM.docx]

**Supplementary Table 1: Possible interactions of BCL-2 family members and BAX/BAK activation**

| Type | Description | Diagram |
| --- | --- | --- |
| Direct activation | The executioners are directly activated by the binding of BH3-only proteins. The availability of these BH3-only proteins for Bax/Bak binding can be controlled by anti-apoptotic proteins. Neither the mitochondrial matrix nor the MOM are necessary for this interaction to be facilitated [[148](#_ENREF_148), [149](#_ENREF_149)]. | BH3-only proteins can directly activate executioners.  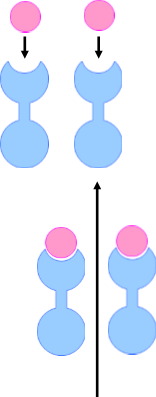  Alternatively, anti-apoptotic proteins can block the BH3 proteins from directly activating the executioners.  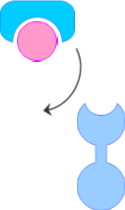 |
| Indirect activation/  derepressor | Although some BH3 proteins are capable of binding to Bax, apoptosis is not dependent on these interactions. Instead, BH3-only proteins interact with anti-apoptotic proteins, sequestering them and thus preventing their inhibition of Bax [[150](#_ENREF_150)]. | Anti-apoptotic protein can block executioner activation, or they can be sequestered by BH3-only proteins.  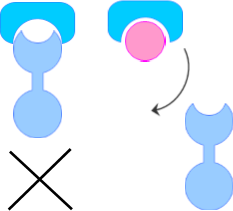 |
| Unified | There are two ‘modes’ of pro-survival activity, wherein the anti-apoptotic proteins have different binding properties. The prevailing mode within a cell can differ depending on cell and stress type. During Mode 1, anti-apoptotic proteins bind BH3 only members, and during Mode 2, they bind Bax/ Bak. Mode 1 is less efficient in supressing apoptosis than Mode 2 [[151](#_ENREF_151)]. | Mode 1: Anti-apoptotic proteins can block executioners.  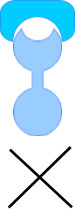  Mode 2: Anti-apoptotic proteins can block BH3-only proteins.  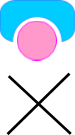 |
| Two-class | The two-class model suggests that the BH3 only Bcl-2 subfamily can be further subdivided into ‘sensitisers’ and ‘activators’. Whereas activators are capable of direct binding to and activation of Bax, sensitizers will bind Bcl-2; Bcl-2 can bind to and sequester the activators and so this sensitiser binding acts to liberate these molecules in a pro-apoptotic manner [[152](#_ENREF_152)]. | Activators (a subtype of BH3-only proteins) can bind to and directly activate executioners.  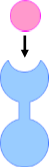  Sensitisers (a subtype of BH3-only proteins) can bind to anti-apoptotic proteins, which stops the anti-apoptotic proteins from sequestering activators.  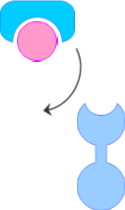 |
| Embedded together | This hypothesis emphasizes the significance of the mitochondrial membrane. Bax and Bak have multiple conformational states which occur in dynamic equilibrium. This equilibrium can be shifted by binding of Bcl-2 family members or other mitochondrial localised proteins, as well as by interactions with the mitochondrial membranes. It is only when Bax/Bak adopt a specific conformation that permeabilisation of the membrane occurs [[153](#_ENREF_153), [154](#_ENREF_154)] | The mitochondrial membrane is vital for all of the interactions of the BCl-2 family.  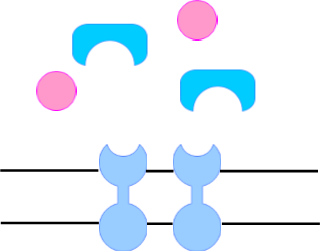 |

(executioners
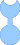
 ; BH3-only proteins
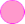
 ; anti-apoptotic proteins
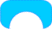
 )
